# Sunflower oil supplementation affects the expression of *miR-20a-5p* and *miR-142-5p* in the lactating bovine mammary gland

**DOI:** 10.1371/journal.pone.0185511

**Published:** 2017-12-27

**Authors:** Lenha Mobuchon, Sandrine Le Guillou, Sylvain Marthey, Johann Laubier, Denis Laloë, Sébastien Bes, Fabienne Le Provost, Christine Leroux

**Affiliations:** 1 GABI, INRA, AgroParisTech, Université Paris-Saclay, Jouy-en-Josas, France; 2 INRA, UMR1213 Herbivores, Saint Genès Champanelle, France; 3 Clermont Université, VetAgro Sup, UMR Herbivores, Clermont-Ferrand, France; University of Illinois, UNITED STATES

## Abstract

Oil supplementation in dairy cattle diets is used to modulate milk fat composition, as well as the expression of mammary lipogenic genes, whose regulation remains unclear. MiRNAs are small non-coding RNA considered as crucial regulators of gene expression, offering clues to explain the mechanism underlying gene nutriregulation. The present study was designed to identify miRNAs whose expression in the cow mammary gland is modulated by sunflower oil supplementation. MiRNomes were obtained using RNAseq technology from the mammary gland of lactating cows receiving a low forage diet, supplemented or not with 4% sunflower oil. Among the 272 miRNAs characterized, eight were selected for RT-qPCR validations, showing the significant down-regulation of *miR-142-5p* and *miR-20a-5p* by sunflower supplementation. These two miRNAs are predicted to target genes whose expression was reported as differentially expressed by sunflower supplementation. Among their putative targets, *ELOVL6* gene involved in lipid metabolism has been studied. However, a first analysis did not show its significant down-regulation, in response to the over-expression of *miR-142-5p*, of *miR-20a-5p*, or both, in a bovine mammary epithelial cell line. However, a clearer understanding of the miRNA expression by lipid supplementation would help to decipher the regulation of lactating cow mammary gland in response to nutrition.

## Introduction

The nutritional quality of milk depends on its composition, and particularly its fatty acid fraction. Modulating the composition of cow's milk is of considerable value since it can have an effect on consumer health. Some fatty acids, such as saturated fatty acids, have been shown to have potentially negative effects on consumer health if eaten in excess, whereas others (e.g. oleic acid, conjugated linoleic acid) have positive effects [1]. Ruminant milk fatty acids can change significantly in response to diet [2]. Nutritional strategies have therefore been developed through dietary supplementation with plant oils or seeds in order to improve the nutritional value of milk [2]. Milk fat synthesis and secretion involve numerous genes whose expression can be modulated by nutrition [3–7]. MicroRNAs (miRNAs), small non-coding RNA with 18–25 nucleotides, are known to be involved in many and various cellular processes [8–10]. They regulate gene expression at the post-transcriptional level by binding to their target mRNA through a base-pairing interaction, mainly inducing translational repression or mRNA degradation [11]. The regulation and function of miRNAs in the mammary gland remain poorly documented, so to gain a clearer understanding of their expression in this organ and their function, profiles have been developed using high throughput sequencing. Several authors have established reference lists of miRNA expressed in the mammary gland of different species, such as human [12], mouse [13], rat [14], pig [15], cow [13,16,17] or goat [18–21]. Recently, *in vitro* studies reported that certain miRNAs, such as *miR-101a* [22] and *miR-126-3p* [23], regulate the proliferation of mammary epithelial cells or, such as *miR-206* [24], impact the development of this organ. In addition, some miRNAs have been reported to be involved in the lactation function, such as *miR-15a* whose over-expression in bovine mammary epithelial cells has been shown to inhibit the expression of beta-casein (*CSN2*), which codes for a major milk protein [25]. Moreover, miRNAs have been evidenced as key regulators of lipid synthesis in various cell lines [26] and tissues [27–29]. In milk lipid synthesis, few studies have indicated their implication [30–32]. The over-expression of *miR-27a* or *miR-103* in goat mammary epithelial cells led to a change in fat droplet and triglyceride accumulation, lowered the unsaturated/saturated fatty acids ratio and markedly affected the expression of genes associated with lipid metabolism [33, 34]. *In vivo* context, studies have shown that the over-expression of *miR-30b* in the mouse mammary gland affects the lipid droplet formation [35]. Taken together, these findings suggest that miRNA may play an important role in mammary gland physiology and function and in milk component synthesis and secretion.

Recent data have suggested that bioactive food components (micro- and macronutrients) affect the expression profile or function of miRNAs [36–40]. For example, in the retroperitoneal adipose tissue of mice, conjugated linoleic acid treatment modified the expression of *miR-103*, *miR-107*, *miR-143*, *miR-221* and *miR-222*, and their expression was correlated with genes that were strongly expressed in adipocytes and related to lipid metabolism [41]. In ruminants, few studies have investigated the impact of nutrition on miRNA expression. Romao and colleagues showed that the expression of eight miRNAs in the backfat and perirenal fat of steers was strongly influenced by a high fat diet [42]. More recently, the expression of 11 miRNAs in the subcutaneous and visceral adipose tissues of lambs was reported to be affected by a change to algae meal from flax oil [43]. Very few data are available on the nutritional regulation of miRNA expression in the mammary gland. An initial nutrigenomics study has reported a modification to the expression of 30 miRNAs by a food restriction in lactating goats [44]. Very recently, Li and colleagues [45] showed that the expression of 14 and 22 miRNAs, in the mammary gland of lactating cows, was affected by linseed and safflower treatments, respectively.

So to gain a clearer understanding of how gene expression in the mammary gland is regulated in response to a diet challenge, the present study aimed to identify miRNAs whose expression was affected by sunflower oil supplementation in lactating cows in relation with transcriptomic data. Thus, we had previously designed an experiment to determine the impact of adding whole and intact rapeseed to a high forage diet, as well as adding sunflower oil to a low forage diet [46]. Only the sunflower supplementation produced a change to gene expression in the mammary gland of cows, associated with an increase of milk production and a decrease of protein and fat content and with greater amplitude of milk composition responses when compared to rapeseed supplementation [46]. Therefore, this diet was selected in order to obtain an overview of the nutritional regulation of milk component biosynthesis by investigating miRNA expression. Two miRNAs, down-regulated by the sunflower oil supplementation, are predicted to target differentially expressed genes previously identified in the same trial. Among their putative targets, *ELOVL6* gene involved in lipid metabolism, has been studied. However, when these miRNAs were transfected to have an over-expression into a bovine mammary epithelial cell line *in vitro*, there was no effect on *ELOVL6* mRNA levels.

## Materials and methods

### Ethics statement and animals

For this study we used samples from an animal experimentation performed at the experimental unit of INRA Research Center of Theix. At the time of the animal experimentation, we did not require to submit each animal trail to ethical committee, we strictly followed the institute’s own severe recommendations of the Animal Care as well as that of the Ethics Committee for Animal Experimentation of the region of Auvergne (CEMEAA number 02). All mammary biopsies, which were the only chirurgical act, were performed with relevant national legislation and were done by an accredited person (N° of certification: 63–20). All collected samples have been previously described [13, 46]. The present study was designed to identify miRNAs whose expression in the cow mammary gland is modulated by sunflower oil supplementation using a high throughput technology. Eleven healthy (standard milk yield (20.7 ± 1.6 kg per day) and without mastitis symptom) multiparous Holstein cows at peak lactation (116.3 ± 8.3 days postpartum at the beginning of the experiment) were offered two experimental diets. The cows were divided into 2 groups based on the homogeneity of their milk yield, age (1,111 ± 55 days) and parity (1.1± 0.3). The eleven cows receiving the two (LF and LF-SO) diets allowing that each cow can be its control. The cows were housed in stalls with free access to water. Each feeding period lasted for 28 days, divided into the first 7 days for treatment adaptation, and the latter 21 days when the cows were fed one of the experimental diets. The diets were: (1) a natural grass hay-based diet plus a mixture of concentrates to obtain an approximate forage-to-concentrate ratio of 46:54, referred to as the “low forage” (LF), and (2) the same LF diet plus 4 g/100g dry matter of sunflower oil (Huilerie de Lapalisse, Lapalisse, France) instead of corn grain (LF-SO). The sunflower oil was mixed and distributed with the forage. The effect of the sunflower oil supplementation on milk product and composition were previously described [46]. Mammary tissues were sampled from the cows at the end of the experimental period by biopsies of the upper one-third of the posterior area of one udder, using the method developed by Farr *et al*. [47]. Approximately 500 mg of mammary tissue were removed and then rinsed in a 0.9% sterile saline solution, inspected to verify tissue homogeneity and snap-frozen in liquid nitrogen.

### Cell transfection

BME-UV1 cells from a mammary epithelial cell line were originally isolated from the mammary gland of a lactating Holstein cow by Zavizion *et al*. [48]. The cells were grown at 37°C under 5% CO2 in a monolayer on plastic culture flasks and in a growth medium described by Zavizion *et al*. [48]. The medium was changed every 48h. To achieve transfection, confluent cells grown in a monolayer were trypsinized with Trypsin-0.25% Ethylenediaminetetraacetic acid (EDTA) and plated in 24-well plate at a rate of 50,000 cells per well. On the following day, 10 pmol of mirVana™ mimic (*hsa-miR-20a-5p*, assay ID MC10057 and *bta-miR-142-5p*, assay ID MC10404) was transfected in triplicate for 24h in the BME-UV1 cells using Lipofectamine® RNAiMAX Reagent (ThermoFisher), according to the manufacturer’s instructions. The mirVana™ miRNA mimic *miR-1-3p* (Ambion™) was used as a positive control and its target TWF1 was quantified to determine whether the mimic was active in the cells. To estimate the transfection rate, the BLOCK-iT™ Alexa Fluor® Red Fluorescent Control (Ambion™) was used on the same plate. Cells were cultured on glass coverslips and transfections were performed at the same time. The cells on coverslips were fixed with 3% paraformaldehyde (freshly prepared in Phosphate buffered saline (PBS) from a 32% stock solution, EMS Hatfield) for 20 min. and maintained at 4°C in PBS until they were mounted on glass slides using VectaShield (Vector) containing DAPI. Images were acquired using a Leica confocal microscope, and cells from three images of three different coverslips were counted. The transfection experiments were repeated four times.

### Isolation of RNA

RNA samples were prepared from 50 mg of mammary tissue using the Nucleospin® miRNA isolation kit (Machery-Nagel, Inc.) as reported by Le Guillou *et al*. [13]. The RNA were precipitated using 3M sodium acetate, 96% ethanol and 5 mg/mL glycogen. For RNA samples prepared from cell cultures, the cells were rinsed with PBS and then RNA were extracted using the RNA Now kit (Ozyme) according to the manufacturer’s instructions and including overnight precipitation so as to guarantee a maximum yield of miRNA. The concentration and purity of RNA were estimated by spectrophotometry (NanodropTH, ND-1000).

### Library preparation, sequencing and data processing

High throughput sequencing was performed on two LF and two LF-SO libraries. The RNA from two cows were pooled to generate each library. The pools were obtained using equal amounts (10 μg) of RNA extracted from the mammary tissues of two cows chosen randomly. The library preparation and sequencing techniques are described in Le Guillou *et al*. [13]. Briefly, libraries were prepared using the Illumina small RNA kit with RNA isolated from mammary gland, followed by sequencing on an Illumina HiSeq 2000 by GATC biotech Company (Next Gen Lab) according to the Solexa sequencing method. The RNA-seq data were then analysed mainly using miRDeep2 software [49] and described in Le Guillou *et al*. [13].

### Reverse transcription and quantitative PCR

The reverse transcription of miRNA was performed on eight miRNA chosen according to their abundance in high throughput sequencing data, their intra-group stability and their fold change (S1 Table): *miR-15a-5p* (TaqMan® ID 005892_mat, Applied Biosystems), *miR-17-5p* (TaqMan® ID 002308), *miR-20a-5p* (TaqMan® ID 00580), *miR-33a-3p* (TaqMan® ID Custom Assay), *miR-126-3p* (TaqMan® ID 008451_mat), *miR-142-5p* (TaqMan® ID 000465), *miR-181a-5p* (TaqMan® ID 000480), *miR-223-3p* (TaqMan® 002295). Reverse transcription and quantitative PCR (qPCR) were performed using the TaqMan® MicroRNA Reverse Transcription and TaqMan® Small RNA Assays (Applied Biosystems), respectively, as described in Mobuchon *et al*. [21].

For gene assays from cell cultures, reverse transcription was performed on 500 ng of total RNA using the SuperScript® VILO cDNA Synthesis kit according to the manufacturer’s instructions (Invitrogen) and under the following conditions: 42°C for 60 min and 85°C for 5 min. Quantitative PCR runs were achieved using ABsolute Blue QPCR Mix, SYBR Green® (Thermo Scientific™) according to the manufacturer’s instructions, on a Mastercycler Ep Realplex system (Eppendorf), under the following conditions: 95°C for 15 min, 45 cycles of 95°C for 15 sec and 60°C for 1 min, and a melting curve. The threshold cycles obtained for *ELOVL6* (F 5’-CAATATTTTCCCAGGGTTCTCC-3’ and R 5’-AGCTGCCCTTTCAAGAGTTG-3’) and *TWF1* (F 5’-GGCATCCAAGCAAGTGAAGA-3’ and R 5’-GCTTCCTACACGACCCAATCA-3’) were normalized with the values of *GAPDH* (GlycerAldehyde-3-Phosphate DeHydrogenase F 5’-ATGGTGAAGGTCGGAGTGAA-3’ and R 5’-ACGATGTCCACTTTGCCAGA-3’), and the results were expressed as fold changes of threshold cycle (Ct) values relative to the control using the 2-ΔΔCt method [50].

### Statistical analysis

Statistical analysis to compare the miRNomes was performed using R version 3.0.1 (R Development Core Team, 2013) with the Bioconductor package DESeq2 [51], as described by Le Guillou *et al*. [13]. Data were filtered using the Bioconductor package HTSFilter [47]. This method aims to identify the threshold that maximizes the filtering similarity among biological replicates, or in other words that where most genes tend to have either normalized counts lower than or equal to the cut-off point in all samples (i.e. filtered genes) or higher than the cut-off point in at least one sample (i.e. non-filtered genes). This threshold value was found to be equal to 199. The p-values were adjusted for multiple testing using the Benjamini-Hochberg method [52], and those with an adjusted p-value <0.1 were considered to be significant.

Quantitative PCR data were analysed with an ANOVA model using R for miRNA TaqMan® Assays. The model included the effects of diet and the random effects of animals. For gene assays, the data were analysed with a Mann-Whitney test using R version 3.0.1. Significance was also declared at a p-value p<0.1.

### miRNA targeted pathways

As seed regions are conserved between human and cattle for *miR-20a-5p* and *miR-142-5p*, putative targets for these miRNA were predicted with a high degree of accuracy based on DIANA-microT-CDS v5.0 [53].

### Availability of data

All milk production and composition data are already published and therefore available in Leroux et al. (2016) [46]. All sequences described in this paper can be downloaded. RNA-seq data from the Hiseq2000 sequencer have been submitted to the GEO repository. They are assigned under the accession number GSE81616.5.

## Results

### Establishment of bovine mammary gland miRNomes

Four libraries were constructed using RNA extracted from the mammary glands of lactating cows fed either a low forage (LF) diet (libraries LF1 and LF2) or the same diet supplemented with 4% sunflower oil (LF-SO, libraries LF-SO1 and LF-SO2) and sequenced using the Next Generation Sequencing (NGS) technology (Table 1). More than 10 million raw reads were obtained for each library. After cleaning the reads (poly-A stretches and adaptors removed), between 77% and 94% of the raw reads were conserved in each library (Table 1). On average, 10,175,799 and 8,578,397 of the size filtered reads (17–28 nucleotides) were mapped on the cow genome (UMD3.1.71) in the LF and LF-SO samples, respectively. Then, 104,286 and 86,752 unique sequences were obtained on average in the LF and LF-SO samples and were processed using miRDeep2 enabling the identification of 1,562 miRNAs.

**Table 1:** Summary of sequencing data. Cows received a low forage diet (LF) or the same diet supplemented with 4% of sunflower oil (LF-SO).

	LF		LF-SO	
	LF1	LF2	LF-SO1	LF-SO2
Raw reads	10,847,425	12,034,673	10,388,132	10,579,276
Clean reads^1^	9,951,849	11,245,052	9,810,954	8,140,532
*% relative to raw data*	*91*.*7%*	*93*.*4%*	*94*.*4%*	*76*.*9%*
Sized reads^2^	9,877,580	11,149,432	9,729,540	8,043,022
*% relative to raw data*	*91*.*1%*	*92*.*6%*	*93*.*7%*	*76%*
Mapped reads	9,549,490	10,802,108	9,456,179	7,700,615
*% relative to sized reads*	*96*.*7%*	*96*.*9%*	*97*.*2%*	*95*.*7%*
Unique sequence	97,136	111,437	85,543	87,971

^1^Poly A stretches and adaptos removed

^2^17-28nt size filtering

HTSFilter package [54] was applied to remove miRNA that appeared to generate an uninformative signal by identifying a filtering threshold that maximizes so-called filtering similarity among replicates. After this filter, the number of miRNAs was reduced to 272 (Supplementary S1 Table). Among these, 18 were predicted miRNAs which were not identified in any species and thus may be considered as potential novel miRNA [13].

### Variations in the bovine mammary gland miRNome as a function of sunflower oil supplementation

**Fig 1:**
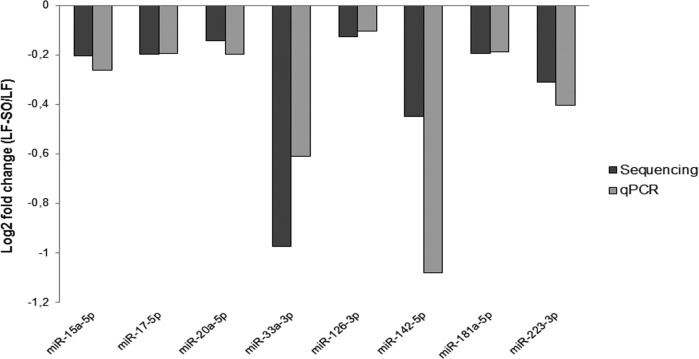
Comparison of the expression of eight miRNA in mammary gland on 2 pools. The analyses were performed on 2 RNA pools from 2 cows received a LF or LF-SO diet obtained by qPCR and NGS approaches. Cows received a low forage diet (LF) or the same diet supplemented with 4% of sunflower oil (LF-SO), n = 2.

**Fig 2:**
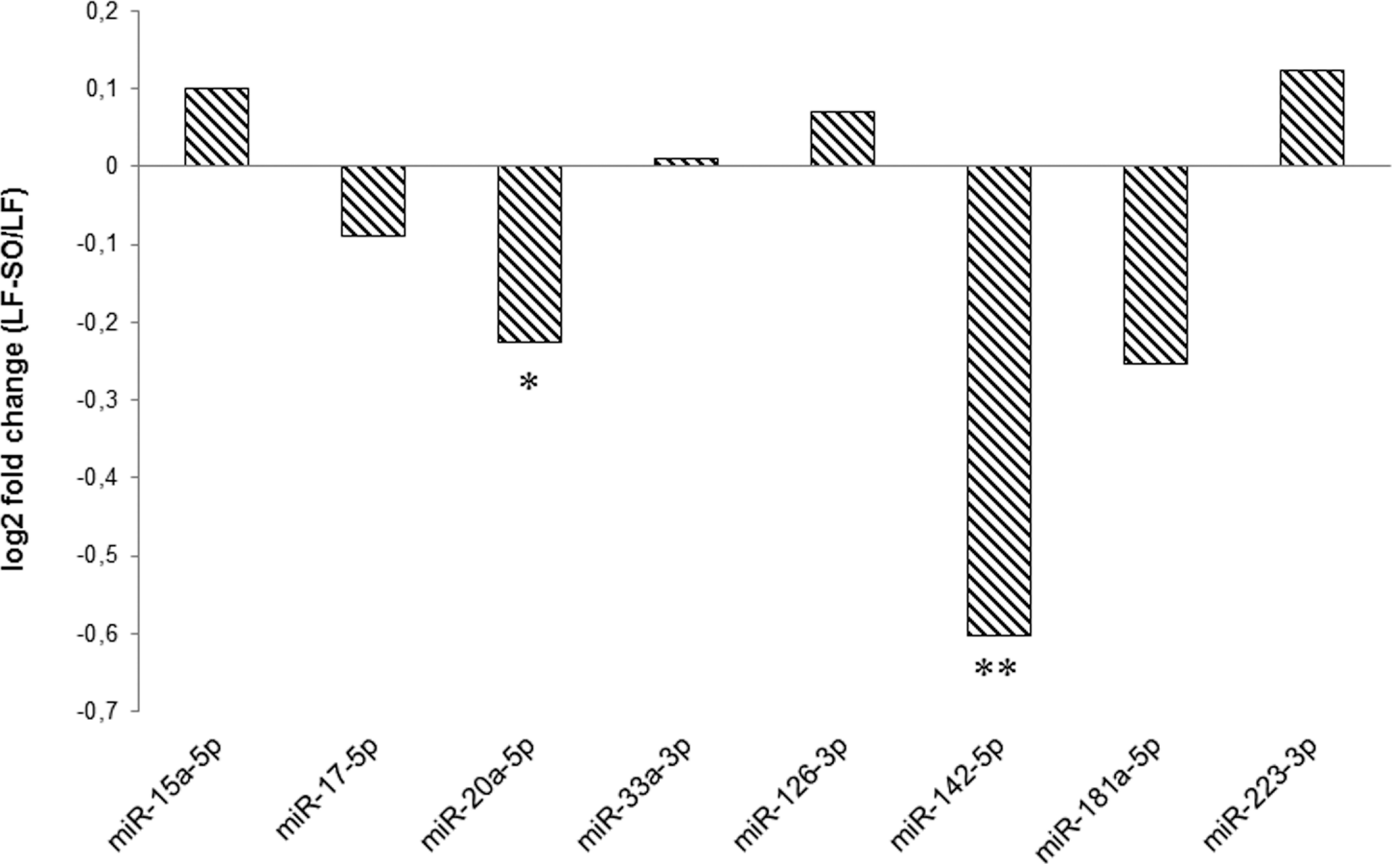
Individual analysis of the expression of eight miRNA in mammary gland. RT-qPCR analyses were performed from 11 cow received a LF or LF-SO diet. Cows received a low forage diet (LF) or the same diet supplemented with 4% of sunflower oil (LF-SO). **0.01<p<0.05, *0.05<p<0.1, n = 11.

This analysis enabled the ranking of miRNAs by their expression level and thus the ranking of their abundance under the two dietary conditions. Eight miRNAs (*miR-15a-5p*, *miR-17-5p*, *miR-20a-5p*, *miR-33a-3p*, *miR-126-3p*, *miR-181a-5p*, *miR-142-5p* and *miR-223-3p*; Supplementary S1 Table) were chosen for further study on the basis of their ranking and their function highlighted in the literature. Their expressions were analysed using qPCR on RNA samples used for sequencing analyses. The profiles obtained using the NGS and qPCR approaches agreed for all the miRNAs tested (Fig 1) despite different amplitude responses. However, with both techniques, the ranking of the expression of the eight selected miRNAs was conserved (data not shown). For example, *miR-126-3p* was the most frequently detected under both the NGS and qPCR approaches, while the expression of *miR-33a-3p* was lower with both techniques when compared to the other miRNA selected. Following the analysis on RNA pools, the expression of the eight miRNAs selected above were validated on a larger number (n = 11) of animals from the same nutritional trial, by RT-qPCR on each individual (Fig 2). Among the eight studied miRNAs, RT-qPCR validations confirmed that *miR-20a-5p* (p = 0.08) and *miR-142-5p* (p = 0.03) were significantly down-regulated by sunflower oil supplementation.

### *miR-20a-5p* and *miR-142-5p* are predicted to target genes differentially expressed by sunflower oil

**Fig 3:**
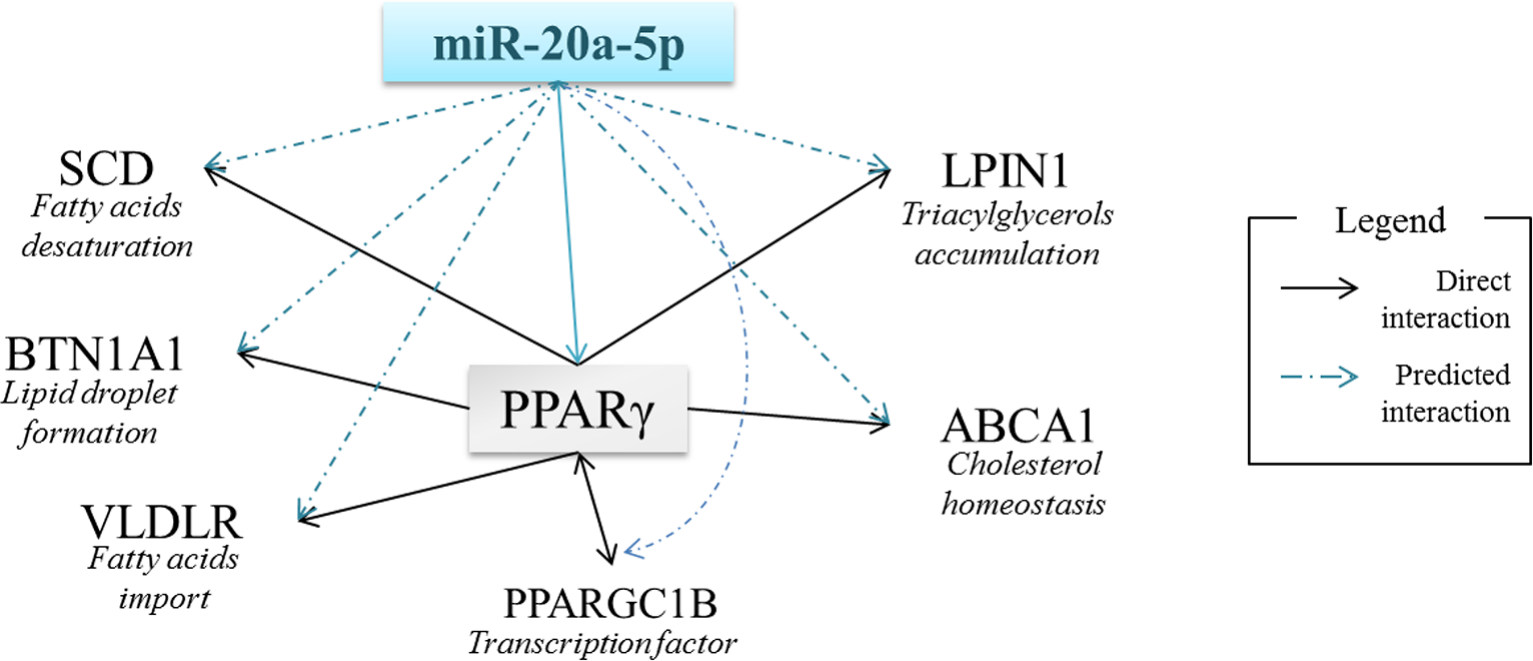
Potential regulations through *miR-20a-5p* in milk fat metabolism. ABCA1: ATP-Binding Cassette subfamily A (ABC1) member1, BTN1A1: BuTyrophiliN subfamily 1, member A1, LPIN1: LiPIN1, PPARγ: Peroxisome Proliferator-Activated Receptor gamma, PPARGC1B: Peroxisome Proliferator-Activated Receptor Gamma Coactivator 1 Beta, SCD: Steroyl-CoA Desaturase, VLDLR: Very Low Density Lipoprotein Lipase Receptor.

In order to investigate the functional role of *miR-20a-5p* and *miR-142-5p*, computational applications were used to predict their targets. Among the hundred potential targets, some genes were involved in lipid metabolism (Table 2) and in common regulatory pathways (Fig 3).

**Table 2:** Predicted targets of *miR-20a-5p* and *miR-142-5p* involved in the lipid metabolism.

miRNA	Gene symbol	Gene name
*miR-20a-5p*	**ABCA1**	**ATP-Binding cassette, sub-family A (ABC1), member 1**
	APOBEC4	Apolipoprotein B mRNA editing enzyme, catalytic polypeptide-like 4 (putative)
	APP	Amyloid beta (A4) precursor protein
	BTN1A1	Butyrophilin, subfamily 1, member 1
	**ELOVL6**	**ELOVL fatty acid elongase 6**
	LDLR	Low density lipoprotein receptor
	LPIN1	Lipin 1
	PPARA	Peroxisome proliferator-activated receptor alpha
	PPARG	Peroxisome proliferator-activated receptor gamma
	**PPARGC1B**	**Peroxisome proliferator-activated receptor gamma, coactivator 1 beta**
	SCD5	Steroyl-CoA desaturase
	VLDLR	Very low density lipoprotein receptor
*miR-142-5p*	**ABCA1**	**ATP-Binding cassette, sub-family A (ABC1), member 1**
	ACADL	Acyl-CoA dehydrogenase, long chain
	ACAT1	Acetyl-CoA acetyltransferase 1
	ACSL1,6	Acyl-CoA synthetase long-chain family member 1,6
	**ELOVL**4, 5, **6**	**ELOVL fatty acid elongase 4,5,6**
	LRP2, 4	Low density lipoprotein receptor related-protein 2, 4
	**PPARGC1B**	**Peroxisome proliferator-activated receptor gamma, coactivator 1 beta**

miRNA’s targets were predicted using Diana microT-CDS (http://diana.imis.athena-innovation.gr/DianaTools/index.php?r=microT_CDS/).

In bold, targets predicted for both miRNA.

**Fig 4:**
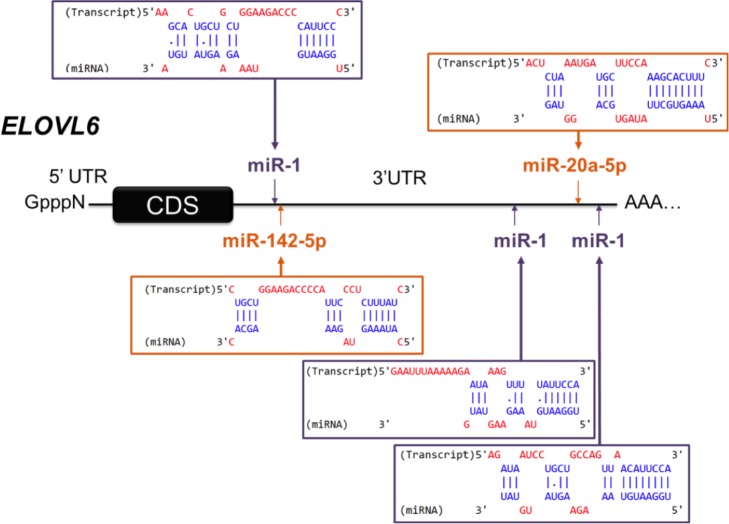
Predicted binding site of *miR-20a-5p*, *miR-142-5p* and *miR-1-3p* in the 3’UTR of *ELOVL6*. Binding sites were predicted with Diana microT-CDS (http://diana.imis.athena-innovation.gr/DianaTools/index.php?r=microT_CDS/).

Differently expressed genes (DEG) on the same samples had previously been detected using transcriptomic analyses [46]. The classification of DEG into functional categories (following Gene Ontology annotation) identified three classes of genes involved in metabolism contained 30% of the DEG, highlighting an effect of sunflower oil on mammary metabolism including the lipid metabolism. Putative targets of *miR-20-5p* and *miR-142-5p* among these DEG were investigated. Among the putative targets displaying inverse responses to treatment compared with miRNA data, nine DEG were highlighted (Table 3). Down-regulated *miR-20a-5p* was found to potentially target eight up-regulated DEG (*ELK4* (ETS transcription factor), *ELOVL6* (fatty acid elongase 6), *ETV1* (ETS variant 1), *KDM6B* (lysine demethylase 6B), *KIAA1524*, *LONP2* (lon peptidase 2, peroxisomal), *M6PR* (mannose-6-phosphate receptor, cation dependent), *USP12* (ubiquitin specific peptidase 12)) in LF-SO cows and *miR-142-5p* was found to potentially target four up-regulated DEG (*ELK4*, *ELOVL6*, *ETV1*, *PIK3CD* (phosphatidylinositol 3-kinase catalytic delta polypeptide)). Both miRNAs potentially targeted *ELK4*, *ETV1* and, in particular, *ELOVL6* gene which encodes for fatty acid elongase, enzyme involved in lipid metabolism (Fig 4).

**Table 3:** Predicted targets of *miR-20a-5p* and *miR-142-5p* among DEG previously identified by microarrays.

analyses [46]. *miRNA*	*Gene symbol*	Gene name	Functional category
*miR-20a-5p*,	*ELK4*	ETS-domain protein	Replication/Transcription/Translation
*miR-142-5p*		(SRF accessory protein 1)	
*miR-20a-5p*,	*ELOVL6*	ELOVL fatty acid elongase 6	Cellular lipid metabolism & transport
*miR-142-5p*			
*miR-20a-5p*,	*ETV1*	ETS variant 1	Replication/Transcription/Translation
*miR-142-5p*			
*miR-20a-5p*	*KDM6B*	Lysine (K)-specific demethylase 6B	Replication/Transcription/Translation
*miR-20a-5p*	*KIAA1524*	KIAA1524 ortholog	Cell cycle,cell growth, proliferation, differentiation & death
*miR-20a-5p*	*LONP2*	Lon peptidase 2n peroxisomal	Cellular protein metabolism & transport
*miR-20a-5p*	*M6PR*	Mannose-6-phophatase receptor (cation dependent)	Cellular carbohydrate metabolism & transport
*miR-20a-5p*	*USP12*	Ubiquitin specific peptidase 12	Cellular protein metabolism & transport
*miR-142-5p*	*PIK3CD*	Phosphoinositide-3-kinase, catalytic, delta polypeptide	Immune, inflammatory and stress response

### Impact of *miR-20a-5p* and *miR-142-5p* on the expression of *ELOVL6*, gene involved in lipid metabolism

**Fig 5:**
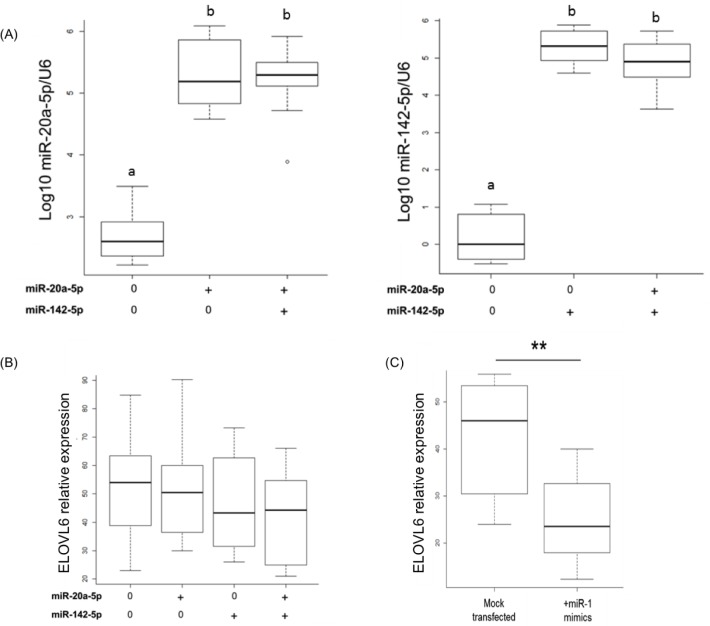
Effect of *miR-20a-5p* and *miR-142-5p* over-expression on *ELOVL6* expression in BME-UV1 cells. (A) Levels of miR-20a-5p and miR-142-5p after transfection with miRNA mimics. Data were obtained by RT-qPCR, normalized regarding U6 values and represented as log10. ELOVL6 expression after transfection of miR-20a-5p and miR-142-5p (B) or miR-1-3p (C). Data were obtained by RT-qPCR, and normalized regarding to GAPDH values. Data are the mean of four transfection experiments. a, b:p<0.01; **:0.01<p<0.05, n = 12.

To investigate whether *miR-20a-5p* and/or *miR-142-5p* could regulate the expression of *ELOVL6* in mammary epithelial cells, miRNAs were over-expressed individually or together using miRNA mimics in culture bovine mammary epithelial monolayers (BME-UV1). The levels of *miR-20a-5p* and *miR-142-5p* were significantly increased in transfected cells when compared with mock-transfected cells (Fig 5A). To estimate the transfection rate, a siRNA labelled with an Alexa Fluor® dye was also transfected in BME-UV1 cells (data not shown). Counting transfected cells *versus* mock-transfected cells enabled to determine a mean transfection rate of 40%. To ensure that the mimics were functional in the cells and able to regulating their target, *miR-1-3p*, a miRNA that regulates the mRNA level of *TWF1* (twinfilin actin binding protein 1) was transfected too. A significant reduction in *TWF1* expression was found in BME-UV1 cells transfected with *miR-1-3p* when compared with mock-transfected cells (data not shown). *MiR-20a-5p* and *miR-142-5p* were transfected separately or together for 48h in BME-UV1 cells, and the expression of *ELOVL6* was quantified by RT-qPCR. No significant changes were obtained in cells transfected with *miR-20a-5p* and *miR-142-5p*, either separately or together (Fig 5B). Furthermore, three binding sites for *miR-1-3p* are also present in the 3’UTR of *ELOVL6* (Fig 4). To ensure the accessibility of *ELOVL6* 3’UTR, the expression of this gene was quantified in both mock-transfected cells and those transfected with *miR-1-3p* mimics. The results revealed a significant decrease of *ELOVL6* in cells transfected with *miR-1-3p* mimics when compared with mock-transfected cells (Fig 5C).

## Discussion

In order to better understand the regulation mechanisms underlying mammary gene expression in response to dietary factors, miRNomes from the mammary glands of cows receiving an LF-SO diet or LF diet were compared. The comparison of these miRNomes enabled identification of several miRNAs that displayed a trend to being up- or down-regulated in the miRNome from cows receiving the LF and LF-SO diets. Among these, eight miRNAs, chosen due to their ranking and their functions, were analysed using qPCR on the same RNA pools. In the same way as several other authors, we show a good correlation between the data obtained using the two different approaches [55, 56], an agreement which reinforced the value of our findings.

Then among the eight candidate miRNAs highlighted by the RNA sequencing study, it was confirmed, by RT-qPCR on samples from 11 cows (each cow receiving each diet), that the expression of *miR-20a-5p* and *miR-142-5p* was decreased by sunflower oil supplementation. The relatively small number of differentially expressed miRNAs in our study could be explained by the duration of the supplementation (21 days). Indeed, Karere *et al*. implemented a seven week challenge in 12 baboons and identified 28 miRNAs that were differentially affected in the liver by a high fat and high cholesterol diet [57]. Furthermore, Romao *et al*. characterized eight miRNAs that were differentially expressed in the adipose tissue of eight steers in response to receiving a high fat diet for 3.5 months [42]. However, a more recent study of 28 days of treatment highlighted 36 miRNAs in the lactating cow mammary miRNome whose expression was modulated by the addition of 5% linseed and 5% safflower oil [45] while no differences were observed after 7 days of treatment. The difference of duration between 21 and 28 days could not be sufficient to explain the difference in terms of the number of differentially expressed miRNAs, but could have been due to the diet used. Indeed, during our study we used two iso-energetic diets, whereas Li *et al*. used oil added to the control diets, leading to a difference in the energy value which could explain the more marked changes in miRNA expression [45]. In addition, we cannot exclude that the relatively small number of differentially expressed miRNAs in our study could be also explained by individual response variations of cows even if each cow was its own control and that we have compared, by RT-qPCR, eleven Holstein mid-lactation cows.

*MiR-20a-5p*, which was differentially expressed in our study, belongs to the *miR-17/92* cluster that has mainly been described for its function as an oncomiR, but is also important to cell cycle proliferation, apoptosis and other biological processes in several organs [58]. Although the response of *miR-20a-5p* to lipid supplementation has not yet been described, the change in the expression of *miR-142-5p* observed here is in line with previous reports regarding adipose tissues. Indeed, in the white adipose tissue of mice receiving a high fat diet for 5 months, *miR-142-5p* was significantly up-regulated when compared with the findings in control mice [59]. Furthermore, its expression was 2.5- and 2.8-fold higher, respectively, in the subcutaneous and visceral fat of lambs receiving 3% algae meal instead of flax oil and barley grain for 140 days [43]. In addition, a high fat diet given in the form of flaxseed to steers for 14 weeks led to 185-fold and 968-fold increases in *miR-142-5p* expression in subcutaneous and visceral adipose tissues, respectively [42]. Both our results and those obtained in adipose tissues suggest that the expression of *miR-142-5p* is strongly influenced by dietary lipids. The opposite variation of the expression observed in the two tissues could be due to the presence of a context of lipid storage in the adipose tissue and a context of lipid secretion in the mammary tissue.

The functions of *miR-20a-5p* and *miR-142-5p* have never been investigated in the mammary gland. Due to the nutritional influences of milk fat composition, in order to determine the effects of sunflower oil supplementation on lipid metabolism which was one of the biological process altered by sunflower oil supplementation [46], our attention focused on the predicted targets involved in this pathway. Among them, twenty-three predicted targets for *miR-20a-5p* and *miR-142-5p* have been identified, using DIANA software, which three were targeted by the two miRNAs. *MiR-20a-5p* may act i) on lipoprotein metabolism (by targeting *APOBEC4* (apolipoprotein B mRNA editing enzyme catalytic polypeptide like 4), *LDLR* (low density lipoprotein receptor) and *VLDLR* (very low density lipoprotein receptor)), ii) on fatty acid desaturation (by targeting *SCD5* (stearoyl-CoA desaturase 5)), and iii) on lipid secretion (by targeting *BTN1A1* (butyrophilin subfamily 1 member A1), a major constituent of the milk fat globule membrane). *LPIN1* (lipin 1) is also potentially targeted by *miR-20a-5p*. This gene is highly expressed in the cow mammary gland at peak lactation and is involved in triacylglycerol accumulation in adipose tissue [60]. In the mammary gland, its role is not yet clear but it could be involved in milk fat synthesis, notably because it is essential for *PPARα* (peroxisome proliferator activated receptor alpha) activation [61]. Members of the PPAR family such as *PPARα*, *PPARγ* and the co-activator *PPARGC1B* (peroxisome proliferative activated receptor, gamma, coactivator 1 beta), are known to be transcription factors involved in the control of genes coding for lipogenic enzymes, are targeted by *miR-142-5p*, and are predicted to be targeted by *miR-20a-5p* [62]. In addition, a direct interaction between *miR-20a-5p* and *PPARγ* has been confirmed using luciferase assay in Cos-7 cells [63]. *PPARγ* controls most of the genes involved in milk fat synthesis, such as *FASN* (fatty acid synthase), *ACACA* (acetyl-CoA carboxylase alpha), *SCD*, *ABCA1*, *BTN1A1* or *LPIN57* (Fig 3). Taken together, these predictions suggest a potential and crucial role for miR-20a-5p at different levels in milk fat synthesis and secretion.

Otherwise, *miR-142-5p* is also predicted to target two isoforms of the *ACSL* (acyl-CoA synthetase long-chain) enzyme (Table 2). *ACSL1* has been shown to be the major isoform in the lactating bovine mammary gland [64], and it activates newly synthesized fatty acids before they can be metabolized or inserted into lipid droplets [65]. Consequently, *miR-20a-5p* and *miR-142-5p* may impact lipid metabolism in response to oil supplementation as a function of their putative targets.

Interestingly, among the predicted targets of *miR-20a-5p* and *miR-142-5p* are nine DEG previously identified in the same samples using previous transcriptomic analysis [46] (Table 3). Both *miR-20a-5p* and *miR-142-5p* are predicted to target *ELK4* and *ETV1* which are known to be involved in replication, transcription and translation. *MiR-20a-5p* also potentially targets *KDM6B*, a demethylase illustrating a mutual regulation of two epigenetic processes [66]. miRNA and DNA methylation are the two epigenetic modifications that have emerged in recent years as the most critical players in the regulation of gene expression. Here, in response to oil supplementation, *miR-20a-5p* will be able to regulate DNA methylation by targeting a demethylase.

Among the nine DEG potentially targeted, four are involved in metabolism one of the biological process identified by transcriptomic analyses and in line with the milk production and milk composition (protein and lipid contents) changes. *LONP2* and *USP12* are part of protein metabolism, whereas *M6PR* plays a role in carbohydrate metabolism. Interestingly, both *miR-20a-5p* and *miR-142-5p* potentially target *ELOVL6*, a member of the family of fatty acid elongases [67], which are involved in lipid metabolism and were over-expressed in our LF-SO samples. It has previously been suggested that ruminant mammary tissue lacks elongases [68], but evidence has pointed to the presence of *ELOVL6* in the mammary gland of mice [69–71] as well as in lactating goat mammary gland [72]. Although its function in mammary gland has not yet been investigated, in the same way as in lipogenic tissues it may catalyze the elongation of saturated and monosaturated fatty acids with 12, 14 and 16 carbons [60]. Further studies are therefore necessary to decipher its role in mammary tissues. It can nevertheless be hypothesized that a weaker expression of *miR-20a-5p* and *miR-142-5p* may have contributed to the increased expression of *ELOVL6* in the mammary glands of cows receiving the LF-SO diet.

To evaluate the role of *miR-20a-5p* and *miR-142-5p*, these miRNAs were over-expressed in bovine mammary epithelial cell line. While miRNAs (alone or together) were highly expressed in transfected cells, they did not lead to a significant reduction in *ELOVL6* expression. However, the over-expression of *miR-1-3p*, which has three putative target sequences in *ELOVL6* 3’UTR, led to a significant reduction in the expression of *ELOVL6*, suggesting that the 3’UTR of this gene is available to miRNA regulation. We can hypothesize that the interaction predicted between *miR-20a-5p* and *miR-142-5p* in BME-UV1 cells does not occur or that the miRNAs affects the protein level. However, the *in vivo* down-regulation of *miR-20a-5p* and *miR-142-5p*, and the up-regulation of *ELOVL6*, were observed in a context of lactation, which was not reproduced in cultured cells where the cellular context may influence miRNA function [73]. Finally, *in vivo*, the link between this gene and these miRNA is perhaps indirect.

Our study thus confirms the effect of lipid supplementation on miRNA expression in the ruminant mammary gland, as observed recently by Li *et al*. [45]. Sunflower oil supplementation altered the expression of *miR-20a-5p* and *miR-142-5p*. These nutriregulated miRNAs potentially target DEG that had previously been identified in the same samples. However, we also showed that one of the DEG potentially targeted by these two miRNA, *ELOVL6*, is not regulated *in vitro*. Deciphering the function of these miRNAs in the mammary gland, and particularly their impact on nutriregulated genes, would be of considerable value to understanding the effects of diet on the regulation of milk synthesis and secretion. In further studies, the regulation of the miRNAs and their potential targets highlighted here will be characterized in other nutrigenomic experimentation to evaluate their involvement in diet modification in general.

## Supporting information

S1 Table MiRNAs NGS data anlysis: Predicted miRNA are in italic and miRNA used for Rt-qPCR are in bold. (PDF)

## References

[1] Shingfield KJ, Chilliard Y, Toivonen V, Kairenius P, Givens DI. Trans fatty acids and bioactive lipids in ruminant milk. Adv Exp Med Biol. 2008; 606: 3–65. doi: 10.1007/978-0-387-74087-4_1 18183924 10.1007/978-0-387-74087-4_1

[2] Chilliard Y, Ferlay A. Dietary lipids and forages interactions on cow and goat milk fatty acid composition and sensory properties. Reprod Nutr Dev. 2004; 44: 467–492. 15636165 10.1051/rnd:2004052

[3] Bernard L, Leroux C, Chilliard Y. Expression and nutritional regulation of lipogenic genes in the ruminant lactating mammary gland. Adv Exp Med Biol. 2008; 67–108. 10.1007/978-0-387-74087-4_2 18183925

[4] Ollier S, Robert-Granié C, Bernard L, Chilliard Y, Leroux C. Mammary trancriptome analysis of food deprived lactating goats highlights genes involved in milk secretion and programmed cell death. J Nutr. 2007; 3: 560–567. 10.1093/jn/137.3.560 17311940

[5] Ollier S, Leroux C, de la Foye A, Bernard L, Rouel J, Chilliard Y. Whole intact rapeseeds or sunflower oil in high-forage or high-concentrate diets affects milk yield, milk composition, and mammary gene expression profile in goats. J Dairy Sci. 2009; 92: 5544–5560. doi: 10.3168/jds.2009-2022 19841217 10.3168/jds.2009-2022

[6] Mach N, van Baal J, Kruijt L, Jacobs A, Smits M. Dietary unsaturated fatty acids affect the mammary gland integrity and health in lactating dairy cows. BMC Proc. 2011; 3: 1753–6561. 10.1186/1753-6561-5-S4-S35 PMC3108231 21645316

[7] Piantoni P, Daniels KM, Everts RE, Rodriguez-Zas SL, Lewin HA, Hurley W, et al Level of nutrient intake affects mammary gland gene expression profiles in preweaned Holstein heifers. J Dairy Sci. 2012; 95: 2550–2561. doi: 10.3168/jds.2011-4539 22541482 10.3168/jds.2011-4539

[8] Ambros V. The functions of animal microRNAs. Nature. 2004; 431: 350–355. doi: 10.1038/nature02871 15372042 10.1038/nature02871

[9] Bushati N, Cohen SM. microRNA functions. Annu Rev Cell Dev Biol. 2007; 23: 175–205. doi: 10.1146/annurev.cellbio.23.090506.123406 17506695 10.1146/annurev.cellbio.23.090506.123406

[10] Ameres SL, Zamore PD. Diversifying microRNA sequence and function. Nat Rev Mol Cell Biol. 2013; 14: 475–488. doi: 10.1038/nrm3611 23800994 10.1038/nrm3611

[11] Bartel DP. MicroRNAs: genomics, biogenesis, mechanism, and function. Cell. 2004; 116: 281–297. 14744438 10.1016/s0092-8674(04)00045-5

[12] Farazi TA, Horlings HM, Ten Hoeve JJ, Mihailovic A, Halfwerk H, Morozov P, et al MicroRNA sequence and expression analysis in breast tumors by deep sequencing. Cancer Res. 2011; 71: 4443–4453. doi: 10.1158/0008-5472.CAN-11-0608 21586611 10.1158/0008-5472.CAN-11-0608 PMC3129492

[13] Le Guillou S, Marthey S, Laloe D, Laubier J, Mobuchon L, Leroux C, et al Characterisation and comparison of lactating mouse and bovine mammary gland miRNomes. PLoS One. 2014; 9: e91938 doi: 10.1371/journal.pone.0091938 24658750 10.1371/journal.pone.0091938 PMC3962357

[14] Zhang C, Zhao Y, Wang Y, Wu H, Fang X, Chen H. Deep RNA sequencing reveals that microRNAs play a key role in lactation in rats. J Nutr. 2014; 144: 1142–1149. doi: 10.3945/jn.114.192575 24899157 10.3945/jn.114.192575

[15] Peng J, Zhao JS, Shen YF, Mao HG, Xu NY. MicroRNA expression profiling of lactating mammary gland in divergent phenotype swine breeds. Int J Mol Sci. 2015; 16: 1448–1465. doi: 10.3390/ijms16011448 25580536 10.3390/ijms16011448 PMC4307312

[16] Li Z, Liu H, Jin X, Lo L, Liu J. Expression profiles of microRNAs from lactating and non-lactating bovine mammary glands and identification of miRNA related to lactation. BMC Genomics. 2012; 13: 731 doi: 10.1186/1471-2164-13-731 23270386 10.1186/1471-2164-13-731 PMC3551688

[17] Li R, Zhang CL, Liao XX, Chen D, Wang WQ, Zhu YH, et al Transcriptome microRNA profiling of bovine mammary glands infected with Staphylococcus aureus. Int J Mol Sci. 2015; 16: 4997–5013. doi: 10.3390/ijms16034997 25749476 10.3390/ijms16034997 PMC4394461

[18] Ji Z, Wang G, Xie Z, Wang J, Zhang C, Dong F, et al Identification of novel and differentially expressed MicroRNAs of dairy goat mammary gland tissues using solexa sequencing and bioinformatics. PLoS One. 2012; 7: 14. 10.1371/journal.pone.0049463 PMC3498112 23166677

[19] Ji Z, Wang G, Xie Z, Zhang C, Wang J. Identification and characterization of microRNA in the dairy goat (Capra hircus) mammary gland by solexa deep-sequencing technology. Mol Biol Rep. 2012; 39: 9361–9371. doi: 10.1007/s11033-012-1779-5 22763736 10.1007/s11033-012-1779-5

[20] Li Z, Lan X, Guo W, Sun J, Huang Y, Fang X, et al Comparative transcriptome profiling of dairy goat microRNAs from dry period and peak lactation mammary gland tissues. PLoS One. 2012; 7: e52388 doi: 10.1371/journal.pone.0052388 23300659 10.1371/journal.pone.0052388 PMC3530564

[21] Mobuchon L, Marthey S, Boussaha M, Le Guillou S, Leroux C, Le Provost F. Annotation of the goat genome using next generation sequencing of microRNA expressed by the lactating mammary gland: comparison of three approaches. BMC Genomics. 2015; 16: 285 doi: 10.1186/s12864-015-1471-y 25888052 10.1186/s12864-015-1471-y PMC4430871

[22] Tanaka T, Haneda S, Imakawa K, Sakai S, Nagaoka K. A microRNA, miR-101a, controls mammary gland development by regulating cyclooxygenase-2 expression. Differentiation. 2009; 77: 181–187. doi: 10.1016/j.diff.2008.10.001 19281778 10.1016/j.diff.2008.10.001

[23] Cui W, Li Q, Feng L, Ding W. MiR-126-3p regulates progesterone receptors and involves development and lactation of mouse mammary gland. Mol Cell Biochem. 2011; 355: 17–25. doi: 10.1007/s11010-011-0834-1 21526342 10.1007/s11010-011-0834-1

[24] Lee MJ, Yoon KS, Cho KW, Kim KS, Jung HS Expression of miR-206 during the initiation of mammary gland development. Cell Tissue Res. 2013; 353: 425–433. doi: 10.1007/s00441-013-1653-3 23733266 10.1007/s00441-013-1653-3

[25] Li HM, Wang CM, Li QZ, Gao XJ. Mir-15a decreases bovine mammary epithelial cell viability and lactation and regulates growth receptor expression. Molecules. 2012; 17: 12037–12048. doi: 10.3390/molecules171012037 23085654 10.3390/molecules171012037 PMC6268530

[26] Esau C, Kang X, Peralta E, Hanson E, Marcusson EG, Ravichandran LV, et al MicroRNA-143 regulates adipocyte differentiation. J Biol Chem. 2004; 279: 52361–52365. doi: 10.1074/jbc.C400438200 15504739 10.1074/jbc.C400438200

[27] Lynn FC. Meta-regulation: microRNA regulation of glucose and lipid metabolism. Trends Endocrinol Metab. 2009; 20: 452–459. doi: 10.1016/j.tem.2009.05.007 19800254 10.1016/j.tem.2009.05.007

[28] Nakanishi N, Nakagawa Y, Tokushige N, Aoki N, Matsuzaka T, Ishii K, et al The up-regulation of microRNA-335 is associated with lipid metabolism in liver and white adipose tissue of genetically obese mice. Biochem Biophys Res Commun. 2009; 385: 492–496. doi: 10.1016/j.bbrc.2009.05.058 19460359 10.1016/j.bbrc.2009.05.058

[29] Aranda JF, Madrigal-Matute J, Rotllan N, Fernandez-Hernando C. MicroRNA modulation of lipid metabolism and oxidative stress in cardiometabolic diseases. Free Radic Biol Med. 2013; 16: 00345–00346. 10.1016/j.freeradbiomed.2013.07.014 PMC4145589 23871755

[30] Wang H, Luo J, Chen Z, Cao WT, Xu HF, Gou DM, et al MicroRNA-24 can control triacylglycerol synthesis in goat mammary epithelial cells by targeting the fatty acid synthase gene. J Dairy Sci. 2015; 14: 00741–00749. 10.3168/jds.2015-9418 26476938

[31] Wang H, Luo J, Zhang T, Tian H, Ma Y, XU H, et al MicroRNA-26a/b and their host genes synergistically regulate triacylglycerol synthesis by targeting the INSIG1 gene. RNA Biol. 2016; 13: 500–510. doi: 10.1080/15476286.2016.1164365 27002347 10.1080/15476286.2016.1164365 PMC4962806

[32] Wang H, Shi H, Luo J, Yi Y, Yao D, Zhang H, et al MiR-145 Regulates Lipogenesis in Goat Mammary Cells Via Targeting INSIG1 and Epigenetic Regulation of Lipid-Related Genes. J Cell Physiol. 2017; 232: 1030–1040. doi: 10.1002/jcp.25499 27448180 10.1002/jcp.25499

[33] Lin XZ, Luo J, Zhang LP, Wang W, Shi HB, Zhu JJ. Mir-27a suppresses triglycerides accumulation and affects gene mRNA expression associated with fat metabolism in dairy goat mammary gland epithelial cells. Gene. 2013; 521: 15–23. doi: 10.1016/j.gene.2013.03.050 23537996 10.1016/j.gene.2013.03.050

[34] Lin X, Luo J, Zhang L, Wang W, Gou D. MiR-103 controls milk fat accumulation in goat (Capra hircus) mammary gland during lactation. PLoS One. 2013; 8: e79258 doi: 10.1371/journal.pone.0079258 24244462 10.1371/journal.pone.0079258 PMC3823599

[35] Le Guillou S, Sdassi N, Laubier J, Passet B, Vilotte M, Castille J, et al Overexpression of miR-30b in the developing mouse mammary gland causes a lactation defect and delays involution. PLoS One. 2012; 7: e45727 doi: 10.1371/journal.pone.0045727 23029204 10.1371/journal.pone.0045727 PMC3454336

[36] Ross SA, Davis CD. MicroRNA, nutrition and cancer prevention. Adv Nutr. 2011; 2: 472–485. doi: 10.3945/an.111.001206 22332090 10.3945/an.111.001206 PMC3226385

[37] Izzotti A, Cartiglia C, Steele VE, De Flora S. MicroRNAs as targets for dietary and pharmacological inhibitors of mutagenesis and carcinogenesis. Mutation Research. 2012; 751: 287–203. doi: 10.1016/j.mrrev.2012.05.004 22683846 10.1016/j.mrrev.2012.05.004 PMC4716614

[38] Shah MS, Davidson LA, Chapkin RS. Mechanistic insights into the role of microRNAs in cancer: influence of nutrient crosstalk. Frontiers in genetics. 2012; 3: 1–14. 23293655 10.3389/fgene.2012.00305 PMC3531809

[39] Garcia-Segura L, Perez-Andrade M, Miranda-Rios J. The Emerging Role of MicroRNAs in the Regulation of Gene Expression by Nutrients. J Nutrigenet Nutrigenomics. 2013; 6: 16–31. doi: 10.1159/000345826 23445777 10.1159/000345826

[40] Milenkovic D, Jude B, Morand C. MiRNA as molecular target of polyphenols underlying their biological effects. Free Radic Biol Med. 2013; 64: 40–51. doi: 10.1016/j.freeradbiomed.2013.05.046 23751562 10.1016/j.freeradbiomed.2013.05.046

[41] Parra P, Serra F, Palou A. Expression of adipose microRNAs is sensitive to dietary conjugated linoleic acid treatment in mice. PLoS One. 2010; 5: e13005 doi: 10.1371/journal.pone.0013005 20886002 10.1371/journal.pone.0013005 PMC2946340

[42] Romao JM, Jin W, He M, McAllister T, Guan LL. Altered microRNA expression in bovine subcutaneous and visceral adipose tissue from cattle under different diet. PLoS One. 2012; 7: e40605 doi: 10.1371/journal.pone.0040605 22815773 10.1371/journal.pone.0040605 PMC3398999

[43] Meale SJ, Romao JM, He ML, Chaves AV, McAllister TA, Guan LL. Effect of diet on microRNA expression in ovine subcutaneous and visceral adipose tissues. J Anim Sci. 2014; 92: 3328–3337. doi: 10.2527/jas.2014-7710 24893997 10.2527/jas.2014-7710

[44] Mobuchon L, Marthey S, Le Guillou S, Laloe D, Le Provost F, Leroux C. Food deprivation affects the miRNome in the lactating goat mammary gland. PLoS One. 2015; 10: e0140111 doi: 10.1371/journal.pone.0140111 26473604 10.1371/journal.pone.0140111 PMC4608672

[45] Li R, Beaudoin F, Ammah AA, Bissonnette N, Benchaar C, Zhao X, et al Deep sequencing shows microRNA involvement in bovine mammary gland adaptation to diets supplemented with linseed oil or safflower oil. BMC Genomics. 2015; 16: 884 doi: 10.1186/s12864-015-1965-7 26519053 10.1186/s12864-015-1965-7 PMC4628385

[46] Leroux C, Bernard L, Faulconnier Y, Rouel J, de la Foye A, Domagalski J, et al Bovine Mammary Nutrigenomics and Changes in the Milk Composition due to Rapeseed or Sunflower Oil Supplementation of High-Forage or High-Concentrate Diets. J Nutrigenet Nutrigenomics. 2016; 9: 65–82. doi: 10.1159/000445996 27318968 10.1159/000445996

[47] Farr VC, Stelwagen K, Cate LR, Molenaar AJ, McFadden TB, Davis SR. An improved method for the routine biopsy of bovine mammary tissue. J Dairy Sci. 1996; 79: 543–549. doi: 10.3168/jds.S0022-0302(96)76398-1 8744218 10.3168/jds.S0022-0302(96)76398-1

[48] Zavizion B, van Duffelen M, Schaeffer W, Politis I. Establishment and characterization of a bovine mammary epithelial cell line with unique properties. In Vitro Cell Dev Biol Anim. 1996; 32: 138–148. 8925136 10.1007/BF02723679

[49] Friedlander MR, Mackowiak SD, Li N, Chen W, Rajewsky N. MiRDeep2 accurately identifies known and hundreds of novel microRNA genes in seven animal clades. Nucleic Acids Res. 2012; 40: 37–52. doi: 10.1093/nar/gkr688 21911355 10.1093/nar/gkr688 PMC3245920

[50] Livak KJ, Schmittgen TD Analysis of relative gene expression data using real-time quantitative PCR and the 2(-Delta Delta C(T)) Method. Methods. 2001 25: 402–408. doi: 10.1006/meth.2001.1262 11846609 10.1006/meth.2001.1262

[51] Anders S, Huber W. Differential expression analysis for sequence count data. Genome Biol. 2010; 11: 2010–2011. 10.1186/gb-2010-11-10-r106 PMC3218662 20979621

[52] Benjamini Y, Hochberg Y. Controlling the false discovery rate: a pratical and powerful approach to multiple testing. J R Statist Soc B. 1995; 57: 289–300.

[53] Paraskevopoulou MD, Georgakilas G, Kostoulas N, Vlachos IS, Vergoulis T, Reczko M, et al DIANA-microT web server v5.0: service integration into miRNA functional analysis workflows. Nucleic Acids Res. 2013; 41: 16. 10.1093/nar/gkt393 PMC3692048 23680784

[54] Rau A, Gallopin M, Celeux G, Jaffrezic F. Data-based filtering for replicated high-throughput transcriptome sequencing experiments. Bioinformatics. 2013; 29: 2146–2152. doi: 10.1093/bioinformatics/btt350 23821648 10.1093/bioinformatics/btt350 PMC3740625

[55] Git A, Dvinge H, Salmon-Divon M, Osborne M, Kutter C, Hadfield J, et al Systematic comparison of microarray profiling, real-time PCR, and next-generation sequencing technologies for measuring differential microRNA expression. RNA. 2010; 16: 991–1006. doi: 10.1261/rna.1947110 20360395 10.1261/rna.1947110 PMC2856892

[56] Tam S, de Borja R, Tsao MS, McPherson JD. Robust global microRNA expression profiling using next-generation sequencing technologies. Lab Invest. 2014; 94: 350–358. doi: 10.1038/labinvest.2013.157 24445778 10.1038/labinvest.2013.157

[57] Karere GM, Glenn JP, VandeBerg JL, Cox LA. Differential microRNA response to a high-cholesterol, high-fat diet in livers of low and high LDL-C baboons. BMC Genomics. 2012; 13: 1471–2164. 10.1186/1471-2164-13-320 PMC3536563 22809019

[58] Mogilyansky E, Rigoutsos I. The miR-17/92 cluster: a comprehensive update on its genomics, genetics, functions and increasingly important and numerous roles in health and disease. Cell Death Differ. 2013; 20: 1603–1614. doi: 10.1038/cdd.2013.125 24212931 10.1038/cdd.2013.125 PMC3824591

[59] Chartoumpekis DV, Zaravinos A, Ziros PG, Iskrenova RP, Psyrogiannis AI, Kyriazopoulou VE, et al Differential expression of microRNAs in adipose tissue after long-term high-fat diet-induced obesity in mice. PLoS One. 2012 7: 4 e34872 doi: 10.1371/journal.pone.0034872 22496873 10.1371/journal.pone.0034872 PMC3319598

[60] Phan J, Reue K. Lipin, a lipodystrophy and obesity gene. Cell Metab. 2005; 1: 73–83. doi: 10.1016/j.cmet.2004.12.002 16054046 10.1016/j.cmet.2004.12.002

[61] Finck BN, Gropler MC, Chen Z, Leone TC, Croce MA, Harris TE, et al Lipin 1 is an inducible amplifier of the hepatic PGC-1alpha/PPARalpha regulatory pathway. Cell Metab. 20106; 4: 199–210. doi: 10.1016/j.cmet.2006.08.005 16950137 10.1016/j.cmet.2006.08.005

[62] Medina-Gomez G, Gray S, Vidal-Puig A. Adipogenesis and lipotoxicity: role of peroxisome proliferator-activated receptor gamma (PPARgamma) and PPARgammacoactivator-1 (PGC1). Public Health Nutr. 2007; 10: 1132–1137. doi: 10.1017/S1368980007000614 17903321 10.1017/S1368980007000614

[63] Zhang JF, Fu WM, He ML, Xie WD, Lv Q, Wan G, et al MiRNA-20a promotes osteogenic differentiation of human mesenchymal stem cells by co-regulating BMP signaling. RNA Biol. 2011; 8: 829–838. doi: 10.4161/rna.8.5.16043 21743293 10.4161/rna.8.5.16043

[64] Bionaz M, Loor JJ. Gene networks driving bovine milk fat synthesis during the lactation cycle. BMC Genomics. 2008; 9: 1471–2164. 10.1186/1471-2164-9-366 PMC2547860 18671863

[65] Mashek DG, McKenzie MA, Van Horn CG, Coleman RA. Rat long chain acyl-CoA synthetase 5 increases fatty acid uptake and partitioning to cellular triacylglycerol in McArdle-RH7777 cells. J Biol Chem. 2006; 281: 945–950. doi: 10.1074/jbc.M507646200 16263710 10.1074/jbc.M507646200

[66] Wang S, Wu W, Claret FX. Mutual regulation of microRNAs and DNA methylation in human cancers. Epigenetics. 2017; 12, 187–197. doi: 10.1080/15592294.2016.1273308 28059592 10.1080/15592294.2016.1273308 PMC5406215

[67] Jakobsson A, Westerberg R, Jacobsson A. Fatty acid elongases in mammals: their regulation and roles in metabolism. Prog Lipid Res. 2006; 45: 237–249. doi: 10.1016/j.plipres.2006.01.004 16564093 10.1016/j.plipres.2006.01.004

[68] Moore JH, Christie WW. Lipid metabolism in the mammary gland of ruminants.; Lipid Metabolism in Ruminant Animals W.W. Christie e, Press, Oxford, UK., editor. 1981. 10.1016/0079-6832(79)90012-0 38463

[69] Padovani M, Lavigne JA, Chandramouli GV, Perkins SN, Barrett JC, Hursting SD, et al Distinct effects of calorie restriction and exercise on mammary gland gene expression in C57BL/6 mice. Cancer Prev Res. 2009; 2: 1076–1087. 10.1158/1940-6207.CAPR-09-0034 PMC2798590 19952363

[70] Rodriguez-Cruz M, Sanchez R, Sanchez AM, Kelleher SL, Sanchez-Munoz F, Maldonado J, et al Participation of mammary gland in long-chain polyunsaturated fatty acid synthesis during pregnancy and lactation in rats. Biochim Biophys Acta. 2011; 4: 284–293. 10.1016/j.bbalip.2011.01.007 21292028

[71] Doria ML, Ribeiro AS, Wang J, Cotrim CZ, Domingues P, Williams C, et al Fatty acid and phospholipid biosynthetic pathways are regulated throughout mammary epithelial cell differentiation and correlate to breast cancer survival. Faseb J. 2014; 28: 4247–4264. doi: 10.1096/fj.14-249672 24970396 10.1096/fj.14-249672

[72] Toral PG, Bernard L, Delavaud C, Gruffat D, Leroux C, Chilliard Y. Effects of fish oil and additional starch on tissue fatty acid profile and lipogenic gene mRNA abundance in lactating goats fed a diet containing sunflower-seed oil. Animal. 2013; 7: 948–956. doi: 10.1017/S1751731113000049 23388097 10.1017/S1751731113000049

[73] Erhard F, Haas J, Lieber D, Malterer G, Jaskiewicz L, Zavolan M, et al Widespread context dependency of microRNA-mediated regulation. Genome Res. 2014; 24: 906–919. doi: 10.1101/gr.166702.113 24668909 10.1101/gr.166702.113 PMC4032855

