# Supplementary material for: Sunflower oil supplementation affects the expression of miR-20a-5p and miR-142-5p in the lactating bovine mammary gland
Source: PLoS One. 2017 Dec 27;12(12):e0185511. doi: 10.1371/journal.pone.0185511 (PMC5744907; doi:10.1371/journal.pone.0185511)
Supplement: S1 Table — (PDF) [file pone.0185511.s001.pdf]

Supplementary table 1

miRNA	Total read counts	Normalized read counts		FC (LF-SO/LF)	padj	Precurseur
		LF	LF-SO			
miR-148a-3p	2792022	671837,3	710421,9	1,06	0,997	bta-mir-148a
let-7a-5p	2593370	608021,7	667701,3	1,10	0,997	bta-let-7a-1
miR-26a-5p	2462913	629487,3	576580,8	0,92	0,997	bta-mir-26a-2
miR-143-3p	2090576	503268,8	532499,4	1,06	0,997	bta-mir-143
miR-21-5p	1885942	474317,2	445517,1	0,94	0,997	bta-mir-21
let-7b-5p	1616561	340061,8	463197,4	1,36	0,997	bta-let-7b
**miR-126-3p**	**1215853**	**311939,5**	**285676,5**	**0,92**	**0,997**	**bta-mir-126**
miR-23a-3p	1170162	267141,6	311237,5	1,17	0,997	bta-mir-23a
miR-200c-3p	964855	247592,2	223567,7	0,90	0,997	bta-mir-200c
miR-16a-5p	925611	236882,2	216616,8	0,91	0,997	bta-mir-16a
miR-22-3p	897291	205064,7	240454,9	1,17	0,997	bta-mir-22
miR-126-5p	872952	221832,0	205280,5	0,93	0,997	bta-mir-126
miR-24-3p	792526	182295,5	210364,3	1,15	0,997	bta-mir-24-1
let-7f-5p	761670	187731,9	185488,2	0,99	0,997	bta-let-7f-2
miR-26b-5p	760794	201567,9	169182,5	0,84	0,997	bta-mir-26b
let-7g-5p	750380	191008,5	176968,9	0,93	0,997	bta-let-7g
miR-141-3p	740441	179397,0	188578,6	1,05	0,997	bta-mir-141
miR-99a-5p	660641	165497,1	158906,1	0,96	0,997	bta-mir-99a
miR-23b-3p	648787	153672,4	165987,6	1,08	0,997	bta-mir-23b
miR-27b-3p	619832	150556,5	155331,6	1,03	0,997	bta-mir-27b
miR-29a-3p	517209	131108,7	122991,6	0,94	0,997	bta-mir-29a
miR-30a-5p	514770	129415,7	124626,8	0,96	0,997	bta-mir-30a
miR-200a-3p	487615	122067,0	118996,0	0,97	0,997	bta-mir-200a
miR-200b-3p	433720	114533,9	96462,1	0,84	0,997	bta-mir-200b
let-7i-5p	413940	98212,6	106434,7	1,08	0,997	bta-let-7i
miR-103-3p	409938	97903,4	105648,9	1,08	0,997	bta-mir-103-1
let-7c-5p	400713	87386,6	110745,3	1,27	0,997	bta-let-7c
**miR-20a-5p**	**398590**	**102734,8**	**93032,6**	**0,91**	**0,997**	**bta-mir-20a**
miR-92a-3p	313042	76760,8	77078,6	1,00	0,997	bta-mir-92a-1
miR-199a-3p	309602	72176,6	80617,0	1,12	0,997	bta-mir-199a-2
miR-27a-3p	274862	65143,9	70873,7	1,09	0,997	bta-mir-27a
miR-186-5p	266167	66110,5	65315,0	0,99	0,997	bta-mir-186
miR-151-5p	251396	62782,9	60962,7	0,97	0,997	bta-mir-151
miR-195-5p	246902	62886,9	58167,8	0,92	0,997	bta-mir-195
miR-10b-5p	244286	64552,5	54412,5	0,84	0,997	bta-mir-10b
miR-29b-3p	231074	61202,0	52222,5	0,85	0,997	bta-mir-29b-2
**miR-181a-5p**	**229522**	**60049,2**	**52490,0**	**0,87**	**0,997**	**bta-mir-181a-2**
miR-145-5p	225561	56449,0	54660,2	0,97	0,997	bta-mir-145
**miR-15a-5p**	**216422**	**56841,0**	**49293,3**	**0,87**	**0,997**	**bta-mir-15a**
miR-16b-5p	205457	53672,2	46804,1	0,87	0,997	bta-mir-16b
miR-497-5p	187051	48192,4	43932,4	0,91	0,997	bta-mir-497
miR-25-3p	180555	45972,7	42341,6	0,92	0,997	bta-mir-25
miR-125a-5p	180142	44514,1	43853,7	0,99	0,997	bta-mir-125a
**miR-17-5p**	**179315**	**47025,5**	**40988,1**	**0,87**	**0,997**	**bta-mir-17**
miR-125b-5p	176697	43104,9	43768,1	1,02	0,997	bta-mir-125b-1
miR-191-5p	158002	43900,2	33071,4	0,75	0,997	bta-mir-191
miR-205-5p	155666	38564,2	37950,0	0,98	0,997	bta-mir-205
miR-30d-5p	150555	36726,0	37507,1	1,02	0,997	bta-mir-30d
miR-320a-3p	134181	31394,8	35246,4	1,12	0,997	bta-mir-320a-2

miR-106b-5p	131501	34703,5	29821,7	0,86	0,997	bta-mir-106b
miR-139-5p	112597	27075,4	28606,3	1,06	0,997	bta-mir-139
let-7d-5p	112435	28714,6	26325,0	0,92	0,997	bta-let-7d
miR-34a-5p	87848	23166,7	19964,4	0,86	0,997	bta-mir-34a
miR-29c-3p	86060	21720,9	20762,0	0,96	0,997	bta-mir-29c
miR-199b-5p	82196	20644,4	19808,1	0,96	0,997	bta-mir-199b
miR-93-5p	81888	19792,0	20649,8	1,04	0,997	bta-mir-93
miR-145-3p	79505	20264,7	18736,5	0,92	0,997	bta-mir-145
miR-375-3p	73826	17923,0	18444,3	1,03	0,997	bta-mir-375
miR-152-3p	73315	17281,0	19027,8	1,10	0,997	bta-mir-152
miR-2284x-5p	72481	17775,3	17961,2	1,01	0,997	bta-mir-2284x
miR-30e-5p	70846	18207,6	16687,3	0,92	0,997	bta-mir-30e
miR-652-3p	70000	16943,4	17629,0	1,04	0,997	bta-mir-652
miR-151-3p	63697	15901,7	15324,5	0,96	0,997	bta-mir-151
miR-423-5p	60894	14171,4	16024,8	1,13	0,997	bta-mir-423
miR-30a-3p	60834	15732,3	14062,1	0,89	0,997	bta-mir-30a
miR-150-5p	59679	15254,5	13793,1	0,90	0,997	bta-mir-150
miR-1468-5p	59190	15499,4	13355,4	0,86	0,997	bta-mir-1468
miR-28-5p	57320	14271,7	13946,6	0,98	0,997	bta-mir-28
miR-424-5p	56261	13588,7	14178,5	1,04	0,997	bta-mir-424
miR-221-3p	53760	13539,5	12937,6	0,96	0,997	bta-mir-221
miR-100-5p	53307	13106,6	13090,6	1,00	0,997	bta-mir-100
miR-374b-5p	51959	12844,9	12524,3	0,98	0,997	bta-mir-374b
miR-181b-5p	43977	11067,3	10592,5	0,96	0,997	bta-mir-181b-2
miR-15b-5p	42099	11345,8	9116,5	0,80	0,997	bta-mir-15b
miR-193a-5p	39436	8716,9	10992,8	1,26	0,997	bta-mir-193a
miR-148b-3p	38939	9852,5	9323,1	0,95	0,997	bta-mir-148b
miR-98-5p	36580	9145,3	8786,8	0,96	0,997	bta-mir-98
miR-451-5p	35102	10280,6	6795,7	0,66	0,997	bta-mir-451
bta-10_2736_mt-3p	*33591*	*7059,9*	*9697,1*	*1,37*	*0,996*	*bta-10_2736*
miR-199c-3p	33045	7742,7	8548,7	1,10	0,997	bta-mir-199c
miR-146a-5p	32712	8191,1	7886,4	0,96	0,997	bta-mir-146a
miR-185-5p	32352	7567,9	8488,1	1,12	0,997	bta-mir-185
miR-429-3p	31621	7853,5	7690,9	0,98	0,997	bta-mir-429
miR-338-3p	31308	7792,2	7703,5	0,99	0,997	bta-mir-338
miR-30e-3p	30958	7963,1	7195,0	0,90	0,997	bta-mir-30e
miR-22-5p	30132	7135,5	7792,0	1,09	0,997	bta-mir-22
miR-340-5p	29168	6986,5	7478,6	1,07	0,997	bta-mir-340
miR-31-5p	28311	6200,6	7914,0	1,28	0,997	bta-mir-31
miR-130a-3p	27314	7270,5	6128,7	0,84	0,997	bta-mir-130a
bta-5_32567_mt-5p	*26612*	*6754,5*	*6316,6*	*0,94*	*0,996*	*bta-5_32567*
miR-362-5p	26155	6586,1	6338,6	0,96	0,997	bta-mir-362
miR-361-5p	25893	6293,6	6466,2	1,03	0,997	bta-mir-361
miR-194-5p	25807	6388,4	6320,3	0,99	0,997	bta-mir-194-1
miR-660-5p	25095	6229,1	6108,7	0,98	0,997	bta-mir-660
miR-18a-5p	24919	6554,9	5672,4	0,87	0,997	bta-mir-18a
miR-101-3p	23742	6433,9	5187,2	0,81	0,997	bta-mir-101-2
miR-1-3p	23544	6703,3	4671,9	0,70	0,997	bta-mir-1-1
miR-342-3p	23485	5889,0	5641,0	0,96	0,997	bta-mir-342
miR-140-3p	23478	6265,9	5252,8	0,84	0,997	bta-mir-140
miR-411a-5p	23402	5144,7	6480,2	1,26	0,997	bta-mir-411a
miR-374a-5p	23134	5956,9	5392,3	0,91	0,997	bta-mir-374a

miR-378-5p	21149	5139,3	5336,9	1,04	0,997	bta-mir-378-2
**miR-142-5p**	**20909**	**5866,4**	**4291,8**	**0,73**	**0,997**	**bta-mir-142**
miR-378-3p	20835	5069,3	5251,0	1,04	0,997	bta-mir-378-1
miR-532-5p	20351	4956,7	5100,7	1,03	0,997	bta-mir-532
miR-146b-5p	19409	5656,3	3631,6	0,64	0,997	bta-mir-146b
miR-450a-5p	19230	4544,6	4951,8	1,09	0,997	bta-mir-450a-2
miR-182-5p	19141	4741,8	4685,4	0,99	0,997	bta-mir-182
miR-106a-5p	18735	4933,8	4290,3	0,87	0,997	bta-mir-106a
miR-214-3p	18295	4041,7	5049,3	1,25	0,997	bta-mir-214
miR-10a-5p	17718	4682,7	3937,3	0,84	0,997	bta-mir-10a
miR-500-5p	17305	4183,3	4384,3	1,05	0,997	bta-mir-500
miR-200b-5p	16471	4093,0	3972,3	0,97	0,997	bta-mir-200b
miR-1839-5p	12455	3251,7	2874,6	0,88	0,997	bta-mir-1839
miR-99b-5p	12409	3025,4	3077,1	1,02	0,997	bta-mir-99b
miR-140-5p	11671	2891,0	2857,1	0,99	0,997	bta-mir-140
miR-425-5p	11642	2903,7	2817,9	0,97	0,997	bta-mir-425
miR-128-3p	11355	2823,8	2765,1	0,98	0,997	bta-mir-128-1
miR-218-5p	11044	2607,8	2828,2	1,08	0,997	bta-mir-218-1
miR-224-5p	10368	2426,8	2694,4	1,11	0,997	bta-mir-224
miR-199a-5p	10060	2460,7	2506,9	1,02	0,997	bta-mir-199a-2
miR-20b-5p	10047	2669,6	2268,1	0,85	0,997	bta-mir-20b
miR-142-3p	9675	2582,4	2143,1	0,83	0,997	bta-mir-142
miR-3431-5p	9039	1986,3	2525,3	1,27	0,997	bta-mir-3431
miR-486-5p	8933	2184,5	2202,1	1,01	0,997	bta-mir-486
miR-423-3p	8266	1907,4	2187,3	1,15	0,997	bta-mir-423
miR-20a-3p	7848	1948,5	1924,1	0,99	0,997	bta-mir-20a
miR-196a-5p	7775	1863,7	1953,1	1,05	0,997	bta-mir-196a-2
miR-196b-5p	7543	1842,7	1873,7	1,02	0,997	bta-mir-196b
bta-16_10094_mt-5p	*7365*	*1851,5*	*1754,5*	*0,95*	*0,996*	*bta-mir-2285t*
miR-155-5p	7164	1913,0	1567,3	0,82	0,997	bta-mir-155
miR-376c-3p	7079	1755,7	1744,8	0,99	0,997	bta-mir-376c
miR-379-5p	7025	1520,8	1975,4	1,30	0,997	bta-mir-379
miR-2284z-5p	6938	1871,3	1512,9	0,81	0,997	bta-mir-2284z-2
bta-16_10667_mt-5p	*6656*	*1469,2*	*1829,0*	*1,24*	*0,996*	*bta-5_31862*
miR-18b-5p	6461	1748,9	1424,6	0,81	0,997	bta-mir-18b
miR-374a-3p	6421	1729,1	1401,4	0,81	0,997	bta-mir-374a
miR-455-3p	6386	1461,8	1708,7	1,17	0,997	bta-mir-455
miR-369-3p	6201	1390,7	1681,9	1,21	0,997	bta-mir-369
miR-106b-3p	5967	1420,1	1535,5	1,08	0,997	bta-mir-106b
miR-17-3p	5958	1553,6	1387,7	0,89	0,997	bta-mir-17
miR-135a-5p	5642	1417,3	1363,2	0,96	0,997	bta-mir-135a-2
miR-222-3p	5615	1372,6	1395,7	1,02	0,997	bta-mir-222
miR-29b-2-5p	5452	1307,4	1394,5	1,07	0,997	bta-mir-29b-2
miR-324-5p	5356	1265,9	1388,7	1,10	0,997	bta-mir-324
miR-183-5p	5296	1232,6	1375,0	1,12	0,997	bta-mir-183
miR-339a-5p	5022	1256,7	1227,8	0,98	0,997	bta-mir-339a
miR-494-3p	4735	1072,5	1278,3	1,19	0,997	bta-mir-494
miR-380-3p	4704	995,5	1349,3	1,36	0,997	bta-mir-380
miR-361-3p	4628	1192,0	1084,1	0,91	0,997	bta-mir-361
miR-7-5p	4548	1165,3	1060,7	0,91	0,997	bta-mir-7-2
miR-1388-3p	4469	1098,7	1096,3	1,00	0,997	bta-mir-1388
miR-32-5p	4438	1166,2	1007,1	0,86	0,997	bta-mir-32

miR-2284ab-5p	4381	1109,4	1037,6	0,94	0,997	bta-mir-2284ab
miR-421-3p	4272	1106,0	983,3	0,89	0,997	bta-mir-421
miR-125b-2-3p	4231	1036,1	1050,0	1,01	0,997	bta-mir-125b-2
miR-376e-3p	4191	1098,9	959,9	0,87	0,997	bta-mir-376e
miR-500a-3p	4013	974,6	1010,5	1,04	0,997	bta-mir-500
miR-499-5p	3980	945,7	1022,9	1,08	0,997	bta-mir-499
miR-542-3p	3862	873,9	1038,4	1,19	0,997	bta-mir-542
miR-30b-5p	3860	963,9	938,6	0,97	0,997	bta-mir-30b
miR-301a-3p	3827	995,5	889,0	0,89	0,997	bta-mir-301a
miR-29a-5p	3730	976,3	848,0	0,87	0,997	bta-mir-29a
miR-30c-2-3p	3655	899,2	905,8	1,01	0,997	bta-mir-30f
miR-96-5p	3554	836,6	926,5	1,11	0,997	bta-mir-96
miR-136-3p	3374	758,9	924,3	1,22	0,997	bta-mir-136
miR-339b-5p	3294	815,5	817,4	1,00	0,997	bta-mir-339b
**miR-223-3p**	**3209**	**869,7**	**697,7**	**0,80**	**0,997**	**bta-mir-223**
miR-6524-3p	2769	706,9	650,1	0,92	0,997	bta-mir-6524
miR-409-3p	2734	512,7	859,2	1,68	0,997	bta-mir-409a
miR-363-3p	2704	671,0	668,8	1,00	0,997	bta-mir-363
miR-487b-3p	2688	590,2	746,4	1,26	0,997	bta-mir-487b
miR-378c-5p	2499	678,2	544,9	0,80	0,997	bta-mir-378c
miR-340-3p	2444	647,6	547,0	0,84	0,997	bta-mir-340
miR-200a-5p	2423	584,1	613,7	1,05	0,997	bta-mir-200a
miR-493-5p	2357	497,4	670,3	1,35	0,997	bta-mir-493
miR-92b-3p	2348	597,8	550,9	0,92	0,997	bta-mir-92b
miR-2284aa-5p	2245	596,9	499,1	0,84	0,997	bta-mir-2284aa-3
miR-2419-5p	2227	577,9	510,6	0,88	0,997	bta-mir-2419
bta-26_24925_mt-3p	*2196*	*502,3*	*580,0*	*1,15*	*0,996*	*bta-26_24925*
miR-376b-3p	2177	621,8	444,5	0,71	0,997	bta-mir-376b
miR-331-5p	2090	513,5	518,2	1,01	0,997	bta-mir-331
miR-484-5p	2060	495,8	524,1	1,06	0,997	bta-mir-484
miR-19b-3p	2032	530,8	467,1	0,88	0,997	bta-mir-19b-2
bta-7_35384_mt-5p	*2017*	*509,5*	*474,5*	*0,93*	*0,996*	*bta-7_35384*
miR-708-5p	2005	464,7	528,2	1,14	0,997	bta-mir-708
miR-2284y-5p	1936	469,4	486,0	1,04	0,997	bta-mir-2284y-5
bta-25_23442_mt-3p	*1868*	*434,3*	*484,5*	*1,12*	*0,996*	*bta-25_23442*
miR-382-5p	1821	368,1	543,3	1,48	0,997	bta-mir-382
miR-30c-5p	1811	459,1	431,1	0,94	0,997	bta-mir-30c
bta-13_7394_mt-3p	*1800*	*435,8*	*446,0*	*1,02*	*0,996*	*bta-13_7394*
miR-1343-3p	1794	404,9	488,1	1,21	0,997	bta-mir-1343
miR-381-3p	1790	381,9	512,8	1,34	0,997	bta-mir-381
bta-22_21141_mt-5p	*1780*	*495,3*	*367,2*	*0,74*	*0,996*	*bta-22_21141*
miR-10b-3p	1776	429,1	447,3	1,04	0,997	bta-mir-10b
miR-127-3p	1772	397,1	486,8	1,23	0,997	bta-mir-127
miR-95-3p	1772	426,6	446,9	1,05	0,997	bta-mir-95
miR-543-3p	1765	335,6	550,4	1,64	0,997	bta-mir-543
miR-299-5p	1763	396,9	480,9	1,21	0,997	bta-mir-299
bta-29_26806_mt-5p	*1758*	*350,2*	*525,6*	*1,50*	*0,996*	*bta-29_26806*
miR-664b-3p	1751	451,1	404,6	0,90	0,997	bta-mir-664b
miR-30b-3p	1713	409,7	433,7	1,06	0,997	bta-mir-30b
miR-365-3p	1705	413,6	429,0	1,04	0,997	bta-mir-365-1
miR-149-5p	1704	379,4	469,8	1,24	0,997	bta-mir-149
miR-29c-5p	1653	421,8	395,1	0,94	0,997	bta-mir-29c

miR-29d-5p	1648	421,3	392,9	0,93	0,997	bta-mir-29d
miR-452-5p	1598	346,6	451,4	1,30	0,997	bta-mir-452
miR-181c-5p	1598	444,7	334,3	0,75	0,997	bta-mir-181c
let-7a-3p	1589	408,5	372,2	0,91	0,997	bta-let-7a-1
miR-411c-5p	1577	387,1	389,0	1,01	0,997	bta-mir-411c
miR-6119-5p	1546	428,2	328,2	0,77	0,997	bta-mir-6119
miR-378a-5p	1507	360,8	387,2	1,07	0,997	bta-mir-378-1
miR-192-5p	1474	369,2	354,7	0,96	0,997	bta-mir-192
miR-24-5p	1442	370,3	337,8	0,91	0,997	bta-mir-24-1
miR-1306-5p	1389	321,8	363,0	1,13	0,997	bta-mir-1306
miR-6120-3p	1365	330,7	344,6	1,04	0,997	bta-mir-6120
bta-6_33860_mt-3p	*1325*	*353,7*	*292,6*	*0,83*	*0,996*	*bta-X_40652*
miR-181a-3p	1302	360,6	271,7	0,75	0,997	bta-mir-181a-1
bta-26_24925_st-5p	*1300*	*270,6*	*375,6*	*1,39*	*0,996*	*bta-26_24925*
miR-191-3p	1279	307,3	324,0	1,05	0,997	bta-mir-191
miR-454-3p	1253	324,7	287,6	0,89	0,997	bta-mir-454
miR-7859-3p	1235	337,1	266,0	0,79	0,997	bta-mir-7859
miR-424-3p	1226	251,8	360,3	1,43	0,997	bta-mir-424
miR-221-5p	1205	311,0	278,0	0,89	0,997	bta-mir-221
miR-125a-3p	1199	265,3	331,8	1,25	0,997	bta-mir-125a
miR-455-5p	1174	268,6	314,2	1,17	0,997	bta-mir-455
miR-744-5p	1157	295,7	276,1	0,93	0,997	bta-mir-744
miR-450b-5p	1140	308,5	245,4	0,80	0,997	bta-mir-450b
miR-29b-1-5p	1124	298,3	249,7	0,84	0,997	bta-mir-29b-1
bta-26_25392_mt-3p	*1108*	*270,1*	*275,2*	*1,02*	*0,996*	*bta-26_25392*
bta-14_8643_mt-5p	*1080*	*303,1*	*218,3*	*0,72*	*0,996*	*bta-14_8643*
miR-181d-5p	1060	267,5	252,5	0,94	0,997	bta-mir-181d
miR-2285t-3p	1055	270,6	246,2	0,91	0,997	bta-mir-2285t
miR-874-3p	1036	254,1	257,5	1,01	0,997	bta-mir-874
miR-671-5p	1032	227,7	287,3	1,26	0,997	bta-mir-671
miR-6529-5p	993	220,3	271,2	1,23	0,997	bta-mir-6529
miR-195a-3p	924	225,3	230,4	1,02	0,997	bta-mir-195
miR-299a-3p	904	222,0	226,0	1,02	0,997	bta-mir-299
bta-8_37539_mt-5p	*894*	*221,6*	*216,4*	*0,98*	*0,996*	*bta-8_37539*
miR-7-1-3p	884	246,4	182,6	0,74	0,997	bta-mir-7-2
miR-130b-3p	877	244,3	185,0	0,76	0,997	bta-mir-130b
bta-26_25421_mt-5p	*855*	*194,6*	*231,1*	*1,19*	*0,996*	*bta-26_25421*
miR-502a-3p	846	230,5	184,1	0,80	0,997	bta-mir-502a-1
miR-503-5p	835	173,4	245,2	1,41	0,997	bta-mir-503
miR-411-3p	835	190,0	224,1	1,18	0,997	bta-mir-411a
miR-32-3p	828	209,4	198,6	0,95	0,997	bta-mir-32
miR-30c-1-3p	827	192,2	217,5	1,13	0,997	bta-mir-30c
miR-27b-5p	826	197,3	211,4	1,07	0,997	bta-mir-27b
miR-665-3p	825	161,3	254,2	1,58	0,997	bta-mir-665
miR-2284n-5p	792	199,8	190,6	0,95	0,997	bta-mir-2284n
miR-127-5p	786	171,1	223,9	1,31	0,997	bta-mir-127
miR-335-5p	782	183,9	203,2	1,10	0,997	bta-mir-335
miR-147-3p	764	181,4	194,3	1,07	0,997	bta-mir-147
miR-139-3p	763	142,4	240,5	1,69	0,997	bta-mir-139
bta-29_27558_mt-5p	*726*	*144,9*	*216,5*	*1,49*	*0,996*	*bta-29_27558*
miR-2299-3p	685	163,8	176,3	1,08	0,997	bta-mir-2299
miR-532-3p	680	174,3	159,1	0,91	0,997	bta-mir-532

miR-379-3p	678	147,4	189,9	1,29	0,997	bta-mir-379
miR-9-5p	609	175,1	118,7	0,68	0,997	bta-mir-9-2
bta-24_23039_mt-5p	*562*	*120,2*	*158,8*	*1,32*	*0,996*	*bta-mir-2285e-2*
miR-491-5p	558	126,6	152,4	1,20	0,997	bta-mir-491
miR-215-5p	421	133,7	65,8	0,49	0,997	bta-mir-215

Predicted miRNA are in italic

*In **bold** miRNA used for Rt-qPCR*
